# Establishment of a Modular Hemodynamic Simulator for Accurate *In Vitro* Simulation of Physiological and Pathological Pressure Waveforms in Native and Bioartificial Blood Vessels

**DOI:** 10.1007/s13239-021-00577-0

**Published:** 2021-09-23

**Authors:** Florian Helms, Axel Haverich, Mathias Wilhelmi, Ulrike Böer

**Affiliations:** 1 Hannover Medical School, Lower Saxony Centre for Biomedical Engineering, Implant Research and Development (NIFE), Stadtfelddamm 34, 30625 Hannover, Germany; 2 Division for Cardiothoracic-, Transplantation- and Vascular Surgery, Hannover Medical School, Hannover, Germany; 3 Department of Vascular- and Endovascular Surgery, St. Bernward Hospital, Hildesheim, Germany

**Keywords:** Hemodynamic simulator, Bioreactor technique, Pressure waveform, Flow conditioning

## Abstract

**Purpose:**

*In vitro* stimulation of native and bioartificial vessels in perfusable systems simulating natural mechanical environments of the human vasculature represents an emerging approach in cardiovascular research. Promising results have been achieved for applications in both regenerative medicine and etiopathogenetic investigations. However, accurate and reliable simulation of the wide variety of physiological and pathological pressure environments observed in different vessels still remains an unmet challenge.

**Methods:**

We established a modular hemodynamic simulator (MHS) with interchangeable and modifiable components suitable for the perfusion of native porcine—(*i.e. the aorta, brachial and radial arteries and the inferior vena cava*) and bioartificial fibrin-based vessels with anatomical site specific pressure curves. Additionally, different pathological pressure waveforms associated with cardiovascular diseases including hyper- and hypotension, tachy- and bradycardia, aortic valve stenosis and insufficiency, heart failure, obstructive cardiomyopathy and arterial stiffening were simulated. Pressure curves, cyclic distension and shear stress were measured for each vessel and compared to ideal clinical pressure waveforms.

**Results:**

The pressure waveforms obtained in the MHS showed high similarity to the ideal anatomical site specific pressure curves of different vessel types. Moreover, the system facilitated accurate emulation of physiological and different pathological pressure conditions in small diameter fibrin-based vessels.

**Conclusion:**

The MHS serves as a variable *in vitro* platform for accurate emulation of physiological and pathological pressure environments in biological probes. Potential applications of the system include bioartificial vessel maturation in cardiovascular tissue engineering approaches as well as etiopathogenetic investigations of various cardiovascular pathologies.

**Supplementary Information:**

The online version contains supplementary material available at 10.1007/s13239-021-00577-0.

## Introduction

In the human body, blood vessels are exposed to a variety of mechanical stimuli including intraluminal blood pressure, cyclic stretch and shear stress, which all are induced by pulsatile blood flow. These forces do not only depend on the position of the vessel in the vascular system, but are also highly variable among patients with different cardiovascular pathologies.

*In vitro* perfusion systems have been developed and successfully used in different fields of cardiovascular research in the past. On the one hand, perfusion systems play an important role in regenerative medicine and tissue engineering approaches, on the other hand, such systems could serve as *in vitro* platforms for etiopathogenetic investigations or drug and medical device testing in the future.^34,37^ It is therefore necessary to establish reliable *in vitro* systems, which not only mimic the various physiological and pathological mechanical environments of the human cardiovascular system accurately, but also allow for the investigation of both bioartificial and native probes. Herein, it is important to notice that the pressure waveform observed in the human vasculature depends on the anatomical position, vascular wall anatomy and vessel diameter of the respective vessel^23^ and is influenced by a variety of different antegrade and retrograde reflection waves which add up to its characteristic and complex form.

*In vitro* simulation of these physiological mechanical environments using pulsatile perfusion systems represents a promising approach in cardiovascular tissue engineering. However, currently used perfusion systems usually fail to accurately mimic the highly complex pressure waveforms of the human vasculature and work with sinusoidal or unspecific pressure curves instead. Although numerous simulators have been developed and great advances have been made in the field of vascular bioreactor technology over the last three decades,^14^ these systems usually focus on absolute pressure values rather than exact emulation of physiological pressure waveforms.^31,32,41,42^ As an example, other groups as well as our own have shown that mechanical stimulation is of pivotal importance for *in vitro* engineering of both the endothelial^15^ and the smooth muscle cell layer^12^ in bioartificial vessels. However, in these preliminary studies unphysiological pressure waveforms were applied due to insufficiently sophisticated bioreactor techniques. This can be seen as a certain limitation of current *in vitro* culture strategies and shows potential for optimization.

On the other hand, systems that have been established specifically for accurate emulation of physiological pressure environments often do not allow implementation of native or bioartificial biological probes,^34^ require cost-intensive computer control^22,24^ or are limited to only one vessel type.^2^

Considering these aspects, accurate and practicable *in vitro* stimulation of different types of native or bioartificial vascular constructs in a modifiable perfusion system is an unmet challenge, yet. Thus, advances in bioreactor technology are required to mimic the natural mechanical environment of native vessels more accurately and, in consequence, potentially maximize the efficiency of *in vitro* stimulation.

Additionally, the option to selectively simulate appropriate pathological flow- and pressure patterns in artificial vessels could allow for the development of 3D *in vitro* study models usable for investigations targeting the etiopathogenesis of cardiovascular diseases.^35^

In conclusion, there is an ongoing need for more accurate and more variable perfusion systems, that facilitate exact stimulation specific for both the anatomical site and the desired physiological or pathological mechanical environment in vascular regenerative medicine as well as in *in vitro* bioanalytics.

We here present a modular hemodynamic simulator that is suitable for mechanical stimulation of both different native vessels and fibrin-based bioartificial vascular constructs. The focus of this work was to simulate the complex pressure waveforms the blood vessels are exposed to in the human body under physiological and pathological conditions with maximal accuracy.

## Methods

**Figure 1:**
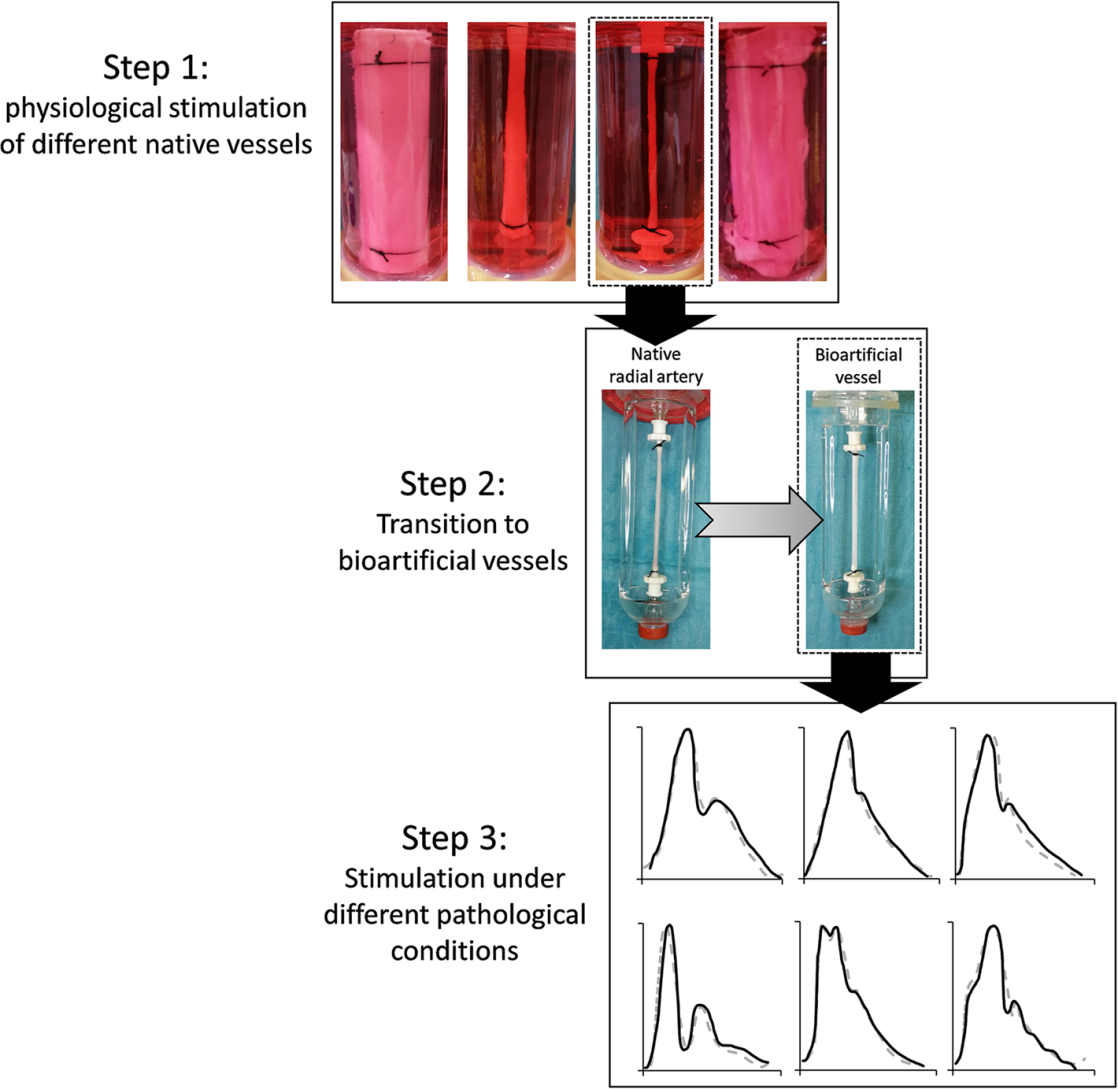
Flow chart. The modular hemodynamic simulator (MHS) was established for the physiological stimulation of different native porcine vessels (step 1). Subsequently, it was adapted to culture small diameter bioartificial vessels under the same physiological conditions as established for the native radial arteries (step 2). Finally, different characteristic pressure waveforms typically associated with common cardiovascular pathologies were simulated in the bioartificial vessels.

The implementation of the MHS followed a three-step approach (Fig. 1). In a first step, the system was calibrated using porcine vessels from different anatomical sites including the descending aorta, brachial and radial arteries as well as the inferior vena cava, which were stimulated with their respective physiological pressure curves in the MHS. The transfer towards bioartificial vessels was performed for the radial artery by comparing the settings of the MHS required for stimulation of either the native vessel or a fibrin-based bioartificial graft. In the third step, various pressure curves associated with characteristic cardiovascular pathologies were generated in the MHS and applied to the bioartificial fibrin vessel.

### Modular Hemodynamic Simulator System Setup

**Figure 2:**
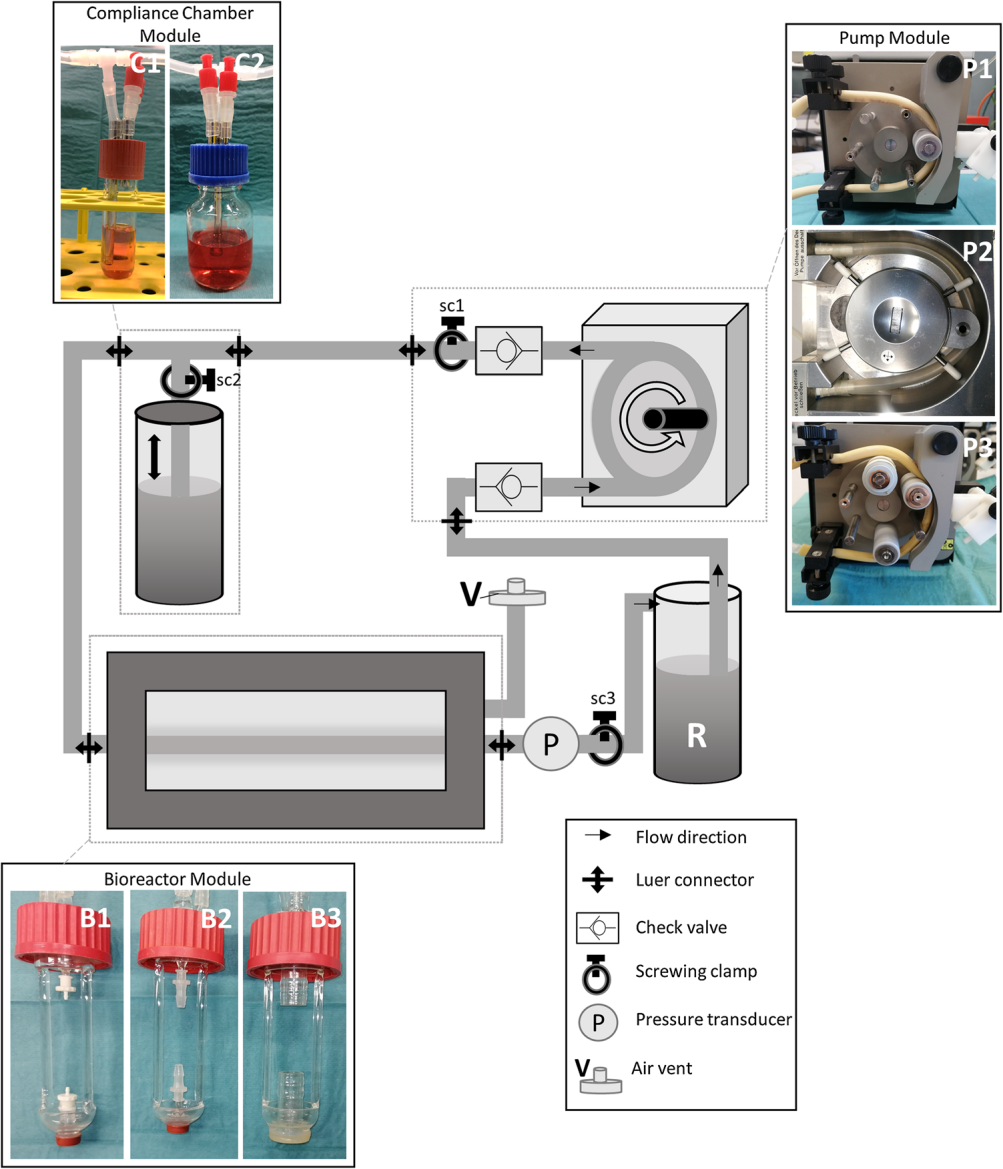
Schematic illustration of the modular perfusion system set up. The different exchangeable modules for the pumps (P1–P3), the compliance chambers (C1, C2) and the bioreactors (B1–B3) were connected via Luer-Lock-connectors. sc1–3 = screwing clamp 1–3.

The MHS consists of three independently interchangeable components: A pump module, a compliance chamber module and a bioreactor module (Fig. 2).

In the pump module, three different pump types were used. A peristaltic pump (Ismatec, Wertheim, Germany; P1) facilitated the perfusion of peripheral small diameter vessels (brachial and radial arteries, bioartificial vessels), whilst a roller pump derived from a heart lung machine (HLM, Stoeckert Shiley, Munich, Germany; P2) was used for the perfusion of the aorta (Table 1). Both pumps were modified by removing rollers to achieve the pulsatile perfusion profile required to mimic the discontinuous mechanical working pattern of the heart. For venous pressure curves, three rollers of different sizes were inserted into the peristaltic pump producing three ejections of different volumes per cycle (P3). Pulse frequency and stroke volume of the pulsatile pumps were adjusted for each perfusion pattern specifically (Tables 1 + 3). Check valves (Buerkle, Bad Bellingen, Germany) upstream and downstream of the pumps imitated the aortic and mitral heart valves respectively and a screwing clamp (sc1) was installed behind the downstream valve to modify the simulated aortic valve orifice area.

**Table 1:** MHS specifications for the simulation of physiological pressure waveforms of different parts of the vasculature.

Vessel type	Pump type	Stroke volume (mL)^†^	Compliance chamber type	Compliance chamber air volume (mL)	Bioreactor type	Circulating volume (mL)
Aorta	P2	7.22 ± 0.16	C2	32	B3	51
Brachial artery	P1	1.18 ± 0.01	C1	9	B2	25
Radial artery	P1	0.87 ± 0.05	C1	15	B1	19
Inferior vena cava	P3	1.34 ± 0.02	C1	10	B3	31

Module types correlate to the items shown in Fig. 2. Pulse frequency was set to 60 bpm for every pulsatile condition

^†^Stroke volume varied within narrow bounds depending on specific afterloads in each vessel. Thus, it is given as mean ± standard deviation

The compliance chamber module was used to imitate the ‘Windkessel’-effect of the aorta and compliant arteries by damping the pressure pulse produced by the pump. For pulsatile perfusion patterns, it was connected to the MHS as a side branch via a T-connector. Its efficiency could be modified firstly by variation of the gas volume using different chamber sizes (C1: total volume: 18 mL, C2: total volume: 66 mL) and variating filling levels and secondly by limitation of volume flow into the chamber using a screwing clamp (sc2). The correlation of compliance chamber air volume and pressure amplitude was determined in a pre-test for different filling levels in both compliance chambers (Fig. S1). To exclude bias due to variable vessel compliance, the pre-test was performed with non-compliant polyvinylchloride-(PVC-)tubes inserted into the bioreactor. The resulting correlation was used as a rough orientation to determine the compliance chamber air volume needed for the targeted pressure amplitude. Fine adjustments of the compliance chamber volume and inflow resistance were set for each physiological and pathological perfusion pattern individually to achieve the targeted pressure waveform and amplitude empirically.

To enable the implementation of different vessel types and diameters (Table 2) into the MHS, three adapters with diameters of 2, 4 and 15 mm were placed into the mounts of the bioreactor module (B1–3). Total volumes of the bioreactors were 103 mL (B1), 102 mL (B2) and 99 mL (B3) respectively. An air vent (V) was placed on the extraluminal component of each bioreactor chamber to allow pressure compensation during cyclic vessel dilatation under pulsatile perfusion. All bioreactors were custom made by the Department of Medical Device Construction at Hannover Medical School (Hannover, Germany).

**Table 2:** Diameters of the native and bioartificial vessels and vascular constructs with the corresponding target pressures for physiological mechanical stimulation in the MHS.

Vessel type	Outer diameter (mm)	Target pressures (mmHg)
Aorta	21.3 ± 0.65	*P*_sys_/*P*_dias_ = 120/80
Brachial artery	3.67 ± 0.2	*P*_sys_/*P*_dias_ = 130/80
Radial artery	2.32 ± 0.1	*P*_sys_/*P*_dias_ = 140/80
Inferior vena cava	19 ± 0.82	*P*_m_ = 2-8

Target pressures comply with physiological pressure ranges of the respective anatomical position in the human body

*P*_sys_ systolic pressure, *P*_dias_ diastolic pressure, *P*_m_ mean pressure

The three modules of the MHS were connected via 1/8″ silicon tubes with Luer-Lock connectors facilitating easy and quick exchange of the different components.

The pressure in the MHS was measured by a pressure transducer (CODAN pvb, Model: DPT-6000; CODAN pvb Critical Care GmbH, Forstinning, Germany), which was connected to the system via Luer-lock with a T-connector. A digital monitor (Datex Ohmeda Corp., Helsinki, Finland) was used to record live pressure waveforms as well as pulse frequency and systolic, diastolic and mean pressures. Averaging during live pressure monitoring was performed in 5 s intervals. After implementation of each new segment, the system was carefully deaired and zero-adjustment was performed. The mean pressure was adapted using a screwing clamp (sc3) and an open reservoir (R) facilitated gas exchange. The whole system except the pump module could be placed into an incubator with 37 °C and 5% CO_2_. Dulbecco’s Modified Eagle Medium (DMEM, Thermo Fischer Scientific, Bremen, Germany) served as perfusion medium.

### Native Vessels and Bioartificial Grafts

Different native vessels were used for the establishment of characteristic pressure curves typical for their anatomical location. Therefore, porcine aortas (*n* = 4), brachial (*n* = 5) and radial arteries (*n* = 3) and inferior venae cavae (*n* = 3) were harvested from German Domestic Pig cadavers (*sus scrofa domesticus*, suidae) under sterile conditions. The descending thoracic aortas were transected distal to the left subclavian artery and mobilized down the level of the diaphragmatic hiatus where they were separated. For the brachial arteries, an approximately 5 cm long segment was excised immediately distal to the continuation of the subclavian artery. The radial arteries were harvested from previously separated forelimbs by transection at the branching point from the median artery and dissection down to the palmar arch. For the inferior vena cava, the segment between the intrahepatic component and the junction to the right atrium was harvested. For transport and storage prior to implementation, all probes were placed in ice-cold phosphate buffered saline (PBS) supplemented with 1% penicillin/streptomycin. To facilitate insertion into the bioreactors, each probe was cut to a length of 5 cm. The respective diameters are shown in Table 2.

**Figure 3:**
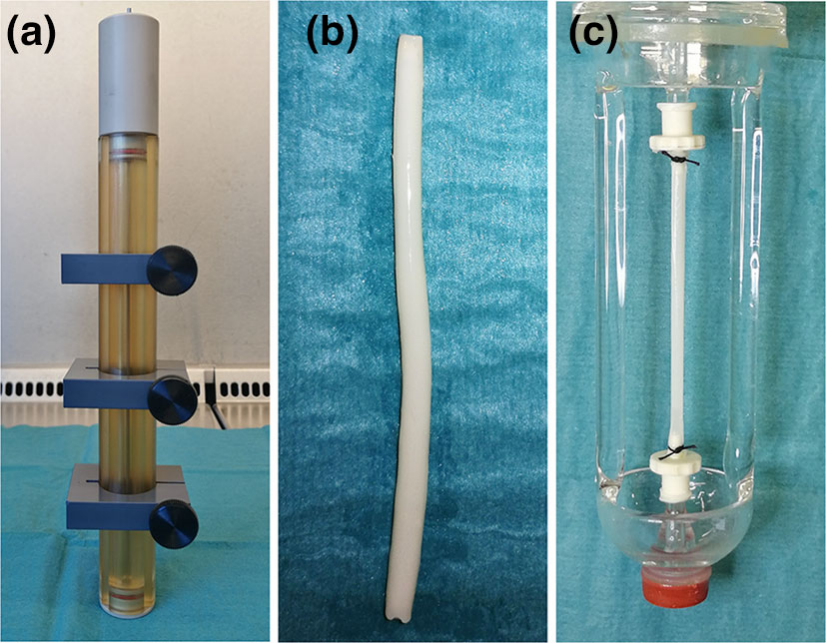
Generation of bioartificial vascular constructs. Small diameter fibrin based vascular grafts were molded in a custom built cylindrical mold (a). After centrifugation and dehydration, constructs were rehydrated, cut to a length of 5 cm (b) and fixed onto the adapters of B1 (c).

**Figure 4:**
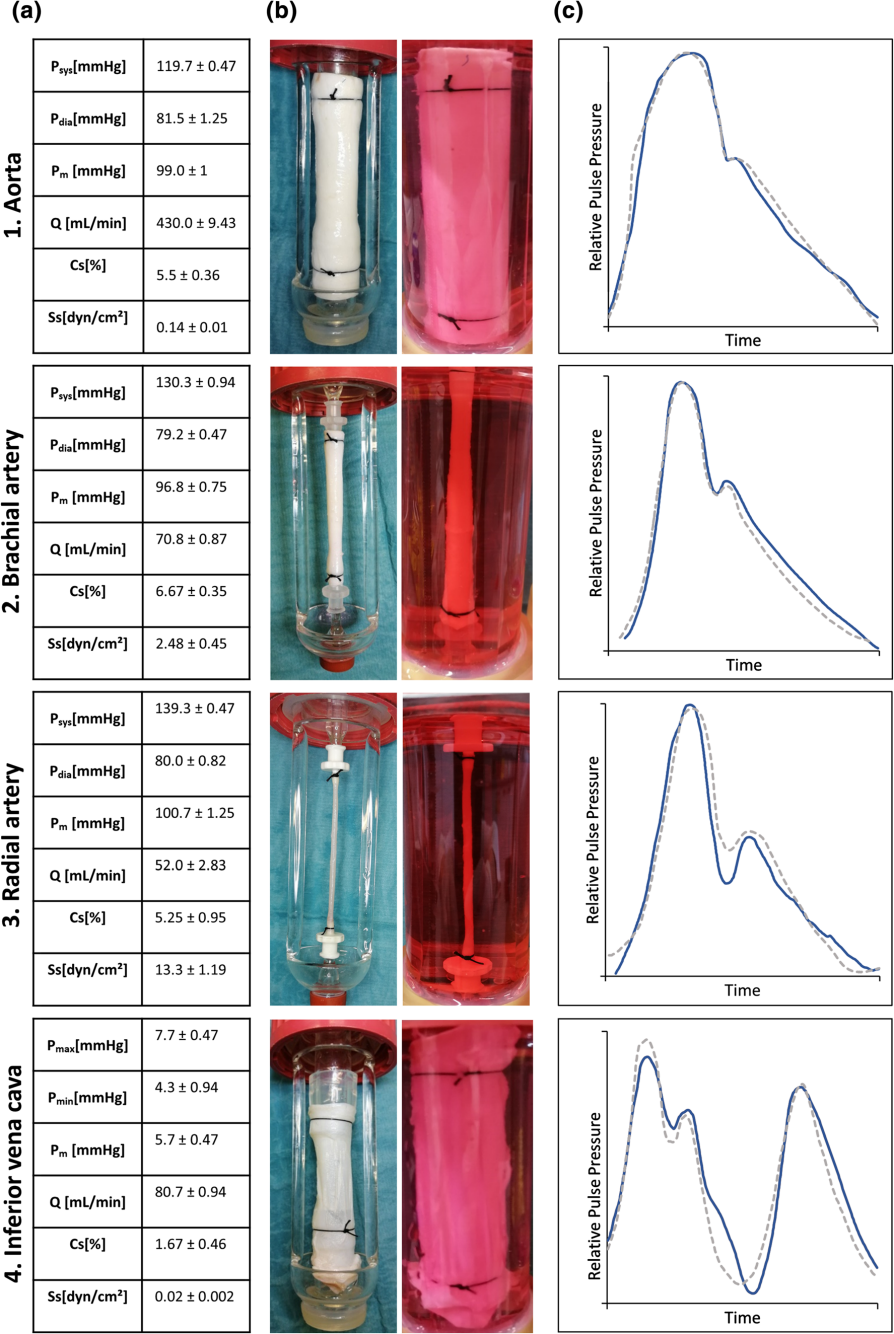
Stimulation of different vessels under anatomical site specific physiological conditions. Different porcine vessels (1: descending aorta, 2: brachial artery, 3: radial artery, 4: inferior vena cava) were implemented into the modular perfusion system that was adapted for the specific vessel type and modified to obtain physiological pressure curves typical for the anatomical position of the respective vessel in the human vasculature. Mechanical parameters (a), vessel morphology after implementation into the bioreactors and during perfusion (b) as well as obtained pressure waveforms (c) are shown. Measured pressure curves (continuous line) and target pressure waveforms adapted from Refs. 3, 13, 18, 27 (dotted line) were normalized to the pulse pressure range (aorta, brachial an radial arteries) or mean pressure (inferior vena cava) to allow qualitative comparison of the obtained MHS-waveform to the ideal waveform by producing an overlay for each vessel type. Absolute pressure values are listed in (A). *P*_sys_ = systolic pressure, *P*_dia_ = diastolic pressure, *P*_max_ = maximum pressure, *P*_min_ = minimum pressure, *P*_m_ = mean pressure, *Q* = volume flow, Cs = cyclic stretch, Ss = shear stress

For transition towards the stimulation of bioartificial vessels, small diameter fibrin-based vascular grafts were generated as described previously.^12^ Briefly, 25 mg cryoprecipitated fibrinogen, 100 U aprotinin (Bayer, Leverkusen, Germany) and 100 *µ*L M199 (Sigma Aldrich, Steinheim, Germany) per graft were replenished by blood serum to 0.5 mL and pH was neutralized by 5 M NaOH. This solution was mixed with 0.5 mL of a solution containing 2.5 U thrombin resolved in 40 mM CaCl_2_ and 2.5 U factor XIII (both from CSL Behring, Marburg, Germany) and injected into a custom build cylindric mold with a luminal placeholder (Fig. 3(a), Department of Medical Device Construction, Hannover Medical School). After 30 min of static polymerization, the bioartificial vessels were compacted by centrifugation in a custom-made rotation unit (Department of Medical Device Construction). Further compaction was achieved by dehydration at 80% humidity for 24 h with subsequent rehydration in PBS for 1 h as described previously.^1^ The resulting bioartificial vessels were 5 cm long with an outer diameter of 2.32 ± 0.03 mm (Fig. 3(b)). The combination of both centrifugation and dehydration methods increased the mechanical stability of the fibrin grafts to a mean burst pressure of 499.8 ± 39.6 mmHg making them suitable for cultivation in the MHS under physiological and supraphysiological mechanical conditions. All vessels were fixated on the adapters of the bioreactors using 2-0 silk ligatures (Figs. 3(c) and 4(b)).

### Mechanical Stimulation

#### Stimulation of Different Native Vessels Under Physiological Conditions

The composition of the MHS was adapted for each vessel type individually (Table 1) to facilitate *in vitro* simulation of the pressure values and waveforms typical for these vessels *in vivo*. The pulse frequency was set to 60 bpm mimicking the physiological basal heart rate. Mean pressures and pulse pressures were adapted to fit the different physiological values throughout the vascular system (Table 2). For the pressure curve of the aorta, the HLM pump (P2) was combined with a large compliance chamber air volume (C2, 32 mL). For the brachial and radial arteries, the smaller peristaltic pump (P1) and smaller compliance chamber volumes (C1) were chosen. Pump P3 was equipped with three rollers of different sizes and combined with a large volume compliance chamber to generate the characteristic central venous pressure waveform for the stimulation of the inferior vena cava.

#### Evaluation of System Reliability

System reliability and reproducibility of the pressure curves were assessed for the stimulation of native brachial arteries. An overlay of 8 consecutive pressure waveforms was generated for cycle-to-cycle consistency. Vessel-to-vessel consistency was evaluated using overlays containing isolated pressure waveforms of 5 different probes. Again, careful deairing and zero-adjustment was performed after implementation of each new probe. The variation in both time and pressure was calculated for the maximum of the dicrotic wave in each setting.

#### Transfer Towards Bioartificial Grafts

The fibrin vessels were implemented in bioreactor B1 in the same way as described for the porcine radial arteries. For both the native radial arteries and the bioartificial graft, the peristaltic pump P1 was combined with compliance chamber C1. The pulse frequency was set to 60 bpm and peripheral pressure curves were adjusted to a target pressure of 140 over 80 mmHg. The remaining system parameters obtained for the native porcine artery were adapted to the mechanical properties of the bioartificial vessel until the target pressure curve was observed.

#### Simulation of Different Pathological Pressure Curves in Bioartificial Vessels

After the transfer was completed and physiological radial artery pressure curves were obtained in bioartificial vessels, different pathological pressure curves were developed by modifications of the MHS modules (Table 3). The pulsatile peristaltic pump (P1) and compliance chamber C1 were used for all conditions and the targeted mean pressure was adapted by modifying the screwing clamp sc3 in each run. Rotation speed of the pump was increased to 120 bpm or decreased to 45 bpm to simulate tachy- and bradycardia respectively. The systemic screwing clamp sc3 was tightened or loosened to increase or decrease mean pressure simulating hyper- and hypotension. The reduction in aortic valve orifice area observed in patients with aortic valve stenosis was mimicked by tightening of the screwing clamp (sc1) directly behind the downstream check valve. Aortic valve insufficiency was simulated by preventing the downstream check valve from closing competently by insertion of a placeholder into the valve canal. Heart failure with severely impaired left ventricular function and reduced ejection fraction was emulated by reduction of the stroke volume of the peristaltic pump. To simulate arterial stiffness which leads to underdamping of the pressure curve, a particularly small compliance chamber air volume was used and to simulate *Pulsus bisferiens*, a waveform frequently observed in patients with hypertrophic obstructive cardiomyopathy, the inflow resistance to the compliance chamber was limited by tightening the corresponding screwing clamp (sc2).

**Table 3:** MHS specifications for the simulation of physiological and different pathological conditions for small diameter fibrin based bioartificial vessels.

Condition	Stroke volume (mL)^†^	Pulse frequency (bpm)	Compliance chamber air volume (mL)	Modifications
Physiological	1.04 ± 0.02	60	11	
Tachycardia	0.87 ± 0.02	**120**	11	Pump speed increased
Bradycardia	1.05 ± 0.02	**45**	11	Pump speed decreased
Hypertension	0.98 ± 0.02	60	10	sc3 tightened
Hypotension	1.03 ± 0.04	60	19	sc3 loosened
Aortic valve stenosis	0.81 ± 0.02	60	11	sc1 tightened
Aortic valve insufficiency	0.97	60	9	Downstream checking valve occlusion impaired
Heart failure	**0.67 ± 0.02**	60	10	Pump stroke volume decreased
Pulsus bisferiens	0.97 ± 0.02	60	19	sc2 tightened
Arterial stiffness	0.97.	60	**4**	Compliance chamber air volume decreased

Numbers and comments in bold represent altered parameters for the specific pathological condition. Here, the same perfusion system build-up consisting of P1, C1 and B1 (Fig. 2) was used for every condition and adapted accordingly to generate the desired condition

*bpm* beats per minute

^†^Stroke volume varied within narrow bounds depending on specific afterloads in each vessel. Thus, it is given as mean ± standard deviation

#### Comparison of ‘In Vitro’ MHS Pressure Curves to Ideal ‘In Vivo’ Waveforms

Pressure curves were digitalized using the software “Engauge Digitizer” (Mark Mitchell, Baurzhan Muftakhidinov and Tobias Winchen *et al.*, "Engauge Digitizer Software."). Both the different physiological and pathological pressure curves obtained in the MHS were compared to ideal pressure curves described for healthy individuals or patients with the respective cardiovascular pathologies. Physiological reference pressure curves were obtained from Kahkashan *et al*.,^18^ Oliver *et al*.,^27^ Huang *et al.*^13^ and Chambers *et al.*^3^ Likewise, characteristic pathological pressure waveforms typically associated with cardiovascular pathologies were obtained from Sabbah *et al*.,^36^ Jenkins *et al*.,^16^ Denardo *et al*.,^5^ Skorton *et al*.^38^ and Nichols *et al.*^26^ For comparison, the ideal pressure curves were digitalized using the “Engauge Digitizer” software as well. Since pressure curves reproduced in basic literature are usually only given as qualitative waveforms without standardized scales for pressure and time, both the measured and the ideal pressure curves had to be normalized to a common scale to facilitate qualitative comparison. For this, the pulse pressure of both the measured and the ideal arterial pressure curves were normalized to the pressure amplitude (diastolic pressure = 0%, systolic pressure = 100%) and cycle length. The central venous pressure curves were normalized to equal mean pressures. Using relative pulse pressures and time intervals allowed the production of overlays and qualitative comparison of measured and ideal pressure curves. Absolute values for the mean, systolic and diastolic pressures were measured for each vessel and condition separately and are given as mean ± standard deviation.

Flow rates were measured volumetrically over a period of 1 min for each run and shear stress was calculated for a pressure-driven laminar flow of an incompressible Newtonian fluid in a cylindrical pipe (Poiseuille flow) using the following formula derived from Hagen-Poiseuille’s law:

$$\text{Shear}\,\text{stress}\,[\text{dyn}/{\text{cm}}^{2}]=\frac{4\mu Q}{\pi {r}^{3}}$$

with *µ* ≙ viscosity of the media, *Q* ≙ cycle-averaged mean volume flow and *r* ≙ baseline radius of the segment.^29^ With this, the cycle-averaged mean wall shear stress over the wall surface of the respective vessels was calculated. To quantify wall strain, video clips of the vessels under each condition were taken and the maximal (‘systolic’) and minimal (‘diastolic’) outer vessel diameters were measured using the software ‘‘MB ruler’’, version 5.3 (Windows Tools) as described previously.^12^ The strain was calculated as follows:

$$\text{Strain}\,\left[\%\right]=\frac{\text{systolic}\,\text{diameter}}{\text{diastolic}\,\text{diameter}}\times 100-100.$$

According to the formula for the circumference $2 r\times \pi$ and assuming a circular cross-section, the change in diameter is directly proportional to the change in circumference.

### Statistics

Statistical analysis was performed using Graphpad Prism 6.04 (Graphpad Software, San Diego, California). For comparisons between two groups, student’s t-test was performed. One Way ANOVA for correlated groups followed by Tukey’s posttest was used to perform comparisons between more than two groups and differences were considered significant at *p* < 0.05.

## Results

### Anatomical Site Specific Physiological Stimulation of Different Native Vessels

Four types of porcine blood vessels were stimulated in the MHS under pressure environments typical for their respective anatomical position in the body. Here, mid-descending aortas served as an example for the application of central pressure waveforms, brachial and radial arteries were used to demonstrate pressure curves in medium-diameter vessels and inferior venae cavae were stimulated under typical central venous pressures. For the aorta, a pulse pressure of 38 mmHg ± 0.82 mmHg resulted in a relative wall strain of 5.5% ± 0.36% and a typical pressure waveform for the mid-descending aorta with a broad maximum and an early incisura^18^ (Fig. 4(1)). Compared to that, the pressure waveform of the brachial artery is characterized by a steeper pressure curve and a more distinct dicrotic wave as well as higher systolic and pulse pressures.^27^ *In vivo*, these effects are due to the lower compliance of the distal arteries which was simulated in the MHS by reducing the compliance chamber volume (Fig. 4(2)). These pressure waveform characteristics are even more pronounced in radial arteries.^13^ In the MHS, a pressure amplitude of 59.3 ± 1.25 mmHg was achieved for the stimulation of native radial arteries which correlated to a cyclic distension of 5.25 ± 0.95% (Fig. 4(3)). The inferior vena cava served as an example of a venous component of the vascular system. The central venous pressure curve is more complex including three maxima (the ‘a wave’ resulting from atrial contraction in end-diastole and the ‘v wave’ resulting from right atrial filling in end-systole as well as a reflection wave ‘c wave’, produced by tricuspid valve closure). This pressure waveform was simulated using three rollers of different sizes in the peristaltic pump (P3) and combining it with a large compliance chamber air volume mimicking the higher compliance of the venous vasculature. The mean central venous pressure (CVP) of 5.7 mmHg ± 0.47 mmHg obtained in the MHS is within the physiological range of 2–8 mmHg and the pressure curve showed the characteristic three maxima and two minima per cycle (Fig. 4(4)).

### Evaluation of Operational Reliability

Pressure curve reproducibility was assessed by producing an overlay of brachial artery pressure curves generated in the MHS (Supplemental Fig. S2). Both cycle-to-cycle and vessel-to-vessel reproducibility was highly satisfying showing overall only small variations which were somewhat greater between different probes compared to those among consecutive cycles. In the vessel-to-vessel comparison, variance in the heights of the incisura and the dicrotic wave was 1.21 mmHg^2^ compared to 0.23 mmHg^2^ in the cycle-to-cycle overlay.

**Figure 5:**
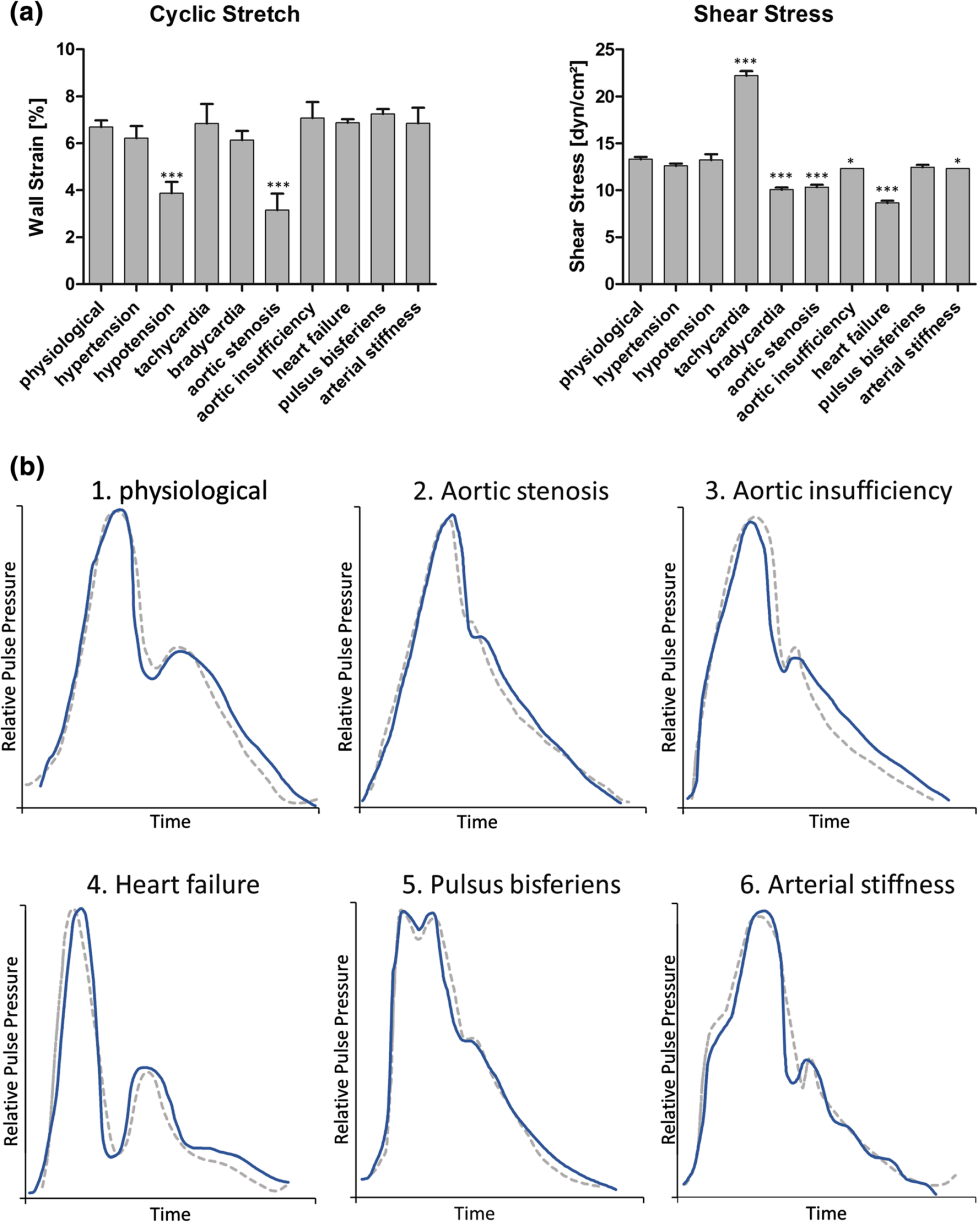
Mechanical patterns obtained from the stimulation of small caliber bioartificial vessels under physiological and pathological conditions. Small diameter fibrin-based bioartificial vessels (n=3 for each condition) were implemented into the modular perfusion system and stimulated under physiological and different pathological mechanical conditions associated with cardiovascular pathologies. (a) Cyclic stretch and shear stress obtained for each condition. Mean values ± standard deviation are shown. The parameters of the pathological stimulation modes were compared to the physiological values by ANOVA. *p < 0.05; ***p < 0.001. (b) Pressure waveform morphologies obtained for different pathological conditions in the MHS. Measured pressure curves (continuous line) and target pressure waveforms adapted from Refs. 5, 13, 16, 26, 36, 38 (dotted line) were normalized to the pulse pressure range to allow qualitative comparison of the obtained MHS-waveform to the ideal clinical waveform by producing an overlay for each condition. The corresponding absolute pressure values are shown in Table 4.

Additionally, the variations of the absolute pressure and shear stress values obtained during physiological and pathological perfusion patterns can serve as indicators for operational reliability. The standard deviation for both systolic and diastolic pressures was <2 mmHg for every physiological and pathological condition (Fig. 4(a), Table 4), for wall strain and shear stress it was < 1% and < 1 dyn/cm^2^ respectively (Fig. 5(a))

**Table 4:** Pulse frequency, pressure and flow values obtained in the MHS for physiological and different pathological mechanical stimulation patterns of small diameter fibrin based vascular grafts.

	Physiological	Tachycardia	Bradycardia	Hypertension	Hypotension	Aortic valve stenosis	aortic valve insufficiency	heart failure	pulsus bisferiens	arterial stiffness
Pf (bpm)	60	120	45	60	60	60	60	60	60	60
*P*_sys_ (mmHg)	139.7 ± 0.47	142.0 ± 0.82	139.3 ± 0.94	165.0 ± 0.82	90.7 ± 0.94	111.3 ± 0.47	139.7 ± 0.94	138.7 ± 2.05	141.0 ± 0.82	140.3 ± 0.94
*P*_dia_ (mmHg)	81.0 ± 0.82	78.0.	81.0 ± 1.41	105.3 ± 1.25	51.3 ± 1.89	81.7 ± 3.40	58.0 ± 0.81	87.3 ± 0.94	71.3 ± 0.94	67.0 ± 1.63
*P*_m_ (mmHg)	99.7 ± 0.47	101.3 ± 2.36	105.0 ± 4.08	125.3 ± 0.47	65.0 ± 1.41	97.3 ± 2.50	86.7 ± 1.70	99.7 ± 0.47	100.7 ± 2.50	96.7 ± 0.47
*Q* (mL/min)	62.7 ± 0.94	104.7 ± 1.89	47.3 ± 0.94	59.3 ± 0.94	62.3 ± 2.36	48.7 ± 0.94	58.0.	40.7 ± 0.94	58.7 ± 0.94	58.0.

Values are given as mean ± standard deviation

*P*_sys_ systolic pressure, *P*_dia_ diastolic pressure, *P*_m_ mean pressure, *Q* volume flow, *Pf* pulse frequency

### Transfer Towards Bioartificial Vessels

After the MHS was established on native vessels, the transfer to the stimulation of bioartificial vascular constructs was performed for the example of the radial artery. For this, fibrin-based vascular constructs with comparable morphological features as observed in native radial arteries were generated. The diameter of the fibrin-based vascular grafts (2.32 mm ± 0.02 mm) was equal to the native radial artery diameter (2.32 mm ± 0.1 mm). Both pressure curves showed the characteristic dicrotic appearance with a steep anacrotic upstroke typical for radial artery waveforms, whereby the dicrotic notch was distinctively lower in the native radial artery group compared to the bioartificial vessels (Figs. 4(c3) and 5(b1)). Both systolic (139.3 vs. 139.7; *p* = 0.52) and diastolic pressures (80.0 vs. 81.0; *p* = 0.29) did not vary significantly between the waveforms of the native and bioartificial vessels. Cyclic stretch was within the physiological range of both vessel types but distinctively higher in fibrin based grafts, although this difference was not statistically significant (6.7 ± 0.23 vs. 5.27 ± 0.94; *p* = 0.16). Both shear stress and wall strain did not differ significantly and were within the physiological range of 10–23 dyn/cm^2^^19^ and 5–10%^17^ respectively for both vessel types (Figs. 4(a3) and 5(a)).

### Simulation of Pathological Pressure Curves in Small Diameter Bioartificial Vessels

In tachy- and bradycardia, volume flow and shear stress were increased or decreased respectively. Pulse pressure was significantly lower under hypotensive conditions leading to decreased wall strain in the vessel (Table 4, Fig. 5(a)).

Compared to physiological conditions, the pulse pressure is also lower in patients with aortic stenosis. Simulation of this phenomenon in the MHS resulted in a significant decrease in cyclic wall strain to 3.2% ± 0.6% (Fig. 5(a)). The main features of the pressure waveform in aortic stenosis including flattening of the anacrotic limb and a small indistinct dicrotic notch^16^ were also present in the waveform obtained in the MHS (Fig. 5(b2)).

Opposed to that, a widened pulse pressure and low diastolic pressure due to backflow through the incompetent valve can be observed in patients with aortic insufficiency.^16^ In the pressure curve this leads to a steep and convex upstroke and a narrow dicrotic notch. In the MHS this correlates to a pulse pressure of 81.7 mmHg ± 1.25 mmHg (Table 4; Fig. 5(b3)).

In severe heart failure with reduced ejection fraction, a characteristic deep dicrotic notch that almost reaches diastolic pressure is found in the pressure curve.^5^ Lowering the stroke volume of the pulsatile pump showed the same effect in the MHS (Fig. 5(b4)). Additionally, this resulted in a decrease of shear stress below physiological levels (8.6 dyn/cm^2^ ± 0.2 dyn/cm^2^). *Pulsus bisferiens* is commonly observed in patients with subaortic left ventricular outflow tract stenosis, possibly due to hypertrophic obstructive cardiomyopathy. The typical waveform including two maxima, a mid-systolic dip and an indistinct dicrotic notch^38^ were simulated in the MHS (Fig. 5(b5)). As arterial stiffness increases by age or progressive arteriosclerosis, pulse pressure increases and a step-like appearance of the anacrotic limb can be observed. In the human body, this so-called 'augmentation pressure' results from a powerful reflection wave, that is propagated more strongly due to incompliant arteries. This phenomenon was simulated in the MHS with a small compliance chamber air volume (Fig. 5(b6)). Slight oscillations were present in the diastolic run off of measured pressure curve. This was most-likely due to underdamping of system-inherent reflection waves and was far less prominent than those observed for the non-compliant mock-vessel during pre-test (Fig. S1-B). Minimal oscillations were also observed under hypertensive conditions.

## Discussion

### Technical Aspects

The MHS presented here can be considered as an attempt on *in vivo* to *in vitro* transition of the human vascular system. Other working groups used two different pumps in their systems by combining a continuous pump for flow administration and a subsequent pulsatile pump for pressure variation.^25,30,33^ This has the advantage that flow and pressure can be regulated relatively independently from each other. In contrast to that, the left ventricle of the heart producing a discontinuous flow and pressure by sequential contractions is represented in the MHS by different modified peristaltic pumps generating both, pulsatile flow and pressure. With this design using only one non-occlusive roller pump, flow rate varied with different afterloads. On the other hand, the more lifelike simulation of the left ventricle of the heart as presented in this study has two major advantages: firstly, the impact of pathologies such as aortic insufficiency and stenosis or heart failure on both flow and pressure can be simulated simultaneously without the need for additional pulsators or flow chambers. In addition to that, using only one pump reduces the MHS size and minimizes perfusion media volume. Moreover, it should be noted that even with two different pumps flow and pressure values are not completely independent from each other since every type of pulsator produces a volume flow itself.

Checking valves were used to simulate the mitral and aortic valve of the human body respectively. Thus, modifications of the downstream checking valve competence and orifice area in the MHS directly translate to the pressure and flow conditions in aortic valve insufficiency or stenosis in patients. This positioning of the checking valves directly upstream and downstream to the pump, which was also used by Thompson *et al.*^40^ allows emulation of the left ventricular pumping pattern. Opposed to that, Buijtenhuijs *et al.* proposed a different valve position with one valve upstream and one valve downstream to the bioreactor module.^7^ In this unphysiological valve position, the check valves can only serve to determine the flow direction but do not contribute to the generation of physiological pressure waveforms. Moreover, simulation of valve pathologies is also only possible, if the valves are inserted in the anatomically correct position as it was implemented in the MHS.

Following blood flow through the body, the first vessel after the aortic valve is the highly compliant aortic arch which assimilates ½ of the stroke volume in each systole and thus contributes the biggest part of the ‘Windkessel’-effect. This is simulated in the MHS using different sized compliance chambers. The theoretical background for the damping effect of the compliance chamber has been studied and described extensively elsewhere.^6,9^ In contrast to other damping systems previously described, which are typically positioned in series connection,^15,30^ the compliance chambers used for the pulsatile perfusion patterns in this MHS are arranged as a side-branch to the main flow system. This arrangement facilitates the simulation of the desired asymmetric pressure curve with a steep upstroke (resulting from a flow fraction bypassing the compliance chamber) and a flatter downstroke (resulting from flow out of the pressurized compliance chamber) observed most clearly in small diameter arterial vessels. In systems using an in-series arrangement of the compliance chamber, this asymmetric pressure curve has to be generated by additional pumps or pulsators downstream of the compliance chamber.^4,11^

The bioreactor module is the centerpiece of the MHS. Due to the modular composition, the MHS could be adapted to implement a variety of different vessels ranging from aortas with an outer diameter of 21.3 mm ± 0.65 mm to small-diameter distal radial arteries with an outer diameter of 2.32 mm ± 0.1 mm. This spectrum of different vessel types exceeds the ranges of previously reported *in vitro* study systems usually focusing on vessels with an inner diameter of 1-4 mm.^2,30^

Using different pump tubes and compliance chambers and combining them as needed for each vessel type individually also led to a reduction of the circulating volume especially for the stimulation of small diameter vessels. Thus, the circulating volume for radial arteries using the smaller pump P1 and compliance chamber C1 was distinctively smaller compared to the volume needed for the stimulation of the aorta with pump P2 and compliance chamber C2 (19 mL vs. 51 mL, Table 1). These circulating volumes are noticeably smaller than total circulation volumes required in comparable perfusion systems published previously.^15,42^ Especially for tissue engineering applications, which often require media supplementation with growth factors and other expensive additives, minimization of circulating media volume is of pivotal importance to increase cost efficacy.

### Anatomical Site Specific Physiological Stimulation of Different Native Vessels

In each anatomical position in the human vascular system, vessels are exposed to a different and unique pressure environment, which is reflected in characteristic pressure waveforms for each vessel type. We established stimulation modes for four different vessel types for accurate emulation of physiological pressure values and pressure waveforms typical for their respective anatomical position in the human body. Until now, MHS used in prior vascular tissue engineering approaches usually generated unphysiological sinusoidal^15^ or unspecific^12,40^ pressure curves. Although promising preliminary results have been achieved with these systems, the optimization of pressure waveforms and adaption to the desired anatomical position as facilitated by the modular MHS could potentially improve the efficacy of *in vitro* mechanical stimulation.

Cyclic stretch, which is mostly influenced by pulse pressure on one hand and compliance of the vessel on the other hand, has been shown to be of pivotal importance especially for the vascular smooth muscle cells of the *tunica media* of the arterial vasculature.^10^ As such, it is an essential parameter for accurate *in vitro* stimulation of vascular constructs. The wall strain achieved in the MHS was within the physiological range for all native arterial constructs. This indicates that the explanted porcine vessels exhibited similar elastic- and stiffness- properties as native human vessels *in vivo*^17,20^ and thus, represent suitable models of the vasculature.

A certain limitation of the MHS can be found with regards to the unphysiologically low wall shear stress obtained in large diameter vessels, because the pump stroke volume was insufficient to generate the extremely high flow rates required when using unmodified standard media. This is mostly due to the low viscosity of the perfusion media (8.9 × 10^−4^ Pa s) compared to the particularly higher blood viscosity (3 × 10^−3^ Pa s). This problem has been reported previously by Tai *et al*., who needed to increase viscosity for *in vitro* stimulation even of small diameter artificial conduits by adding dextran to the perfusion media and thereby achieved physiological shear stress.^39^ Opposed to that, in our study the wall shear stress obtained in radial arteries and small diameter bioartificial vessels were within the physiological range of the respective anatomical position in the human body. Nonetheless, such media modifications can be potentially useful when targeting physiological shear stress in large-diameter vessels. This however would exceed the scope of the present work focusing on physiological pressure rather than flow emulation. For this application, the stroke volume generated by the different pump types was sufficient to generate the desired pressure alternations.

### System Reliability

One of the most important requirements of any *in vitro* BPS is the system reliability and reproducibility of desired mechanical conditions. Here, only slight cycle-to-cycle and vessel-to-vessel variations in the pressure waveforms of the brachial arteries were observed (Supplemental Fig. S2). While the cycle-to-cycle variations indicate system-inherent variations, the stronger vessel-to-vessel variations are most likely due to differences in mechanical properties of the implemented arteries such as luminal diameter (Table 2) and compliance of the vascular wall.

### Transfer Towards Bioartificial Vessels

After the transfer from native radial arteries to bioartificial vascular constructs, the characteristic features of the pressure waveforms were observed in both vessels. The distinctively lower dicrotic notch observed in the native artery is most likely due to higher arterial stiffness compared to the fibrin based graft, which could not be completely compensated by the bigger compliance chamber air volume applied for the former.

### Simulation of Pathological Pressure Curves in the MHS

A variety of different pathological pressure waveforms associated with cardiovascular pathologies were simulated in the MHS and compared to ideal clinical waveforms observed in patients. Although certain differences are noted, the key features of each clinical pathological waveform were present in the measured pressure curve of the MHS. Furthermore, the impact of altered perfusion conditions on cyclic wall strain and shear stress could be observed in the MHS.

*In vitro* simulation of pathological pressure curves is the domain of so called “simulators of cardiovascular loops” (SCVL).^28,43^ However, these systems do not include native or bioartificial probes and focus on the physics of flow and pressure dynamics under different physiological, pathological and mechanical support conditions. As such, they are optimized to accurately produce desired pressure waveforms. Simulation of different pressure waveforms could be performed with a comparable accuracy in the MHS presented here as in the SCVL published by Ruiz *et al.*^34,^ Thus, the *in vitro* simulation of the pressure curves in the presented system is comparable to that of the ‘gold standard’ represented by SCVLs. Since the MHS allows the implementation of native or bioartificial vessels, it is not limited to hemodynamic studies, but potentially useful for tissue engineering and biomedical approaches. Thus, it can represent an *in vitro* platform for the investigation of the impact of different pathological flow patterns on native or bioengineered cells and tissues.

### Limitations

Although variable and accurate perfusion was successfully demonstrated in the MHS, certain limitations of this study need to be considered. First, pressure and flow cannot be modulated independently from each other in this setup, whereby the screwing clamps used for pressure modulation cause a non-linear increase in resistance at increasing flow rates. This potentially complicates fine-adjustments of flow and pressure at high flow rates. Second, the focus of this study was on the accurate emulation of physiological and pathological pressure environments in straight vessel segments. However, when targeting physiological flow conditions, especially in the simulation of more complex vascular structures with varying luminal diameters, focal and temporal variations in wall shear stress need to be taken into account as etiologically relevant factors of vascular pathologies.^8,21^ In this case, shear stress has to be monitored more accurately and cannot be averaged over the vessel wall area and pressure cycle as performed in this study. Therefore, modifying media viscosity to achieve physiological shear stress in large-caliber vessels and live-monitoring of flow and focal shear stress need to be considered in the future.

## Conclusion and Future Applications

We here presented a modular hemodynamic simulator for *in vitro* stimulation of native and bioartificial vascular constructs under anatomical site specific physiological and pathological mechanical conditions. Compared to previous systems, the complexity of pressure curves observed in healthy individuals and patients with cardiovascular pathologies can be emulated with higher accuracy and variability in the MHS. The main features of the system are: (i) The composition of the MHS from independently interchangeable modules allows easy rearrangement and enables the simulation of a broad spectrum of aortic, arterial and venous pressure environments. (ii) Specific modifications of the characteristics of each module facilitate fine adjustments of physiological pressure curves as well as accurate simulation of different pathological pressure waveforms typical for specific cardiovascular diseases and (iii) usage of size-adjusted modules for each application minimizes the circulating media volume and increases the efficiency of the MHS.

The system additionally facilitates reproducible and lifelike *in vitro* stimulation of bioartificial vessels. This is of interest for tissue engineering approaches and potentially increases the efficacy of *in vitro* mechanical stimulation. The option to implement a variety of different vessel types and selectively stimulate vascular constructs under pathological conditions can be a first step in the development of an *in vitro* platform for etiopathogenetic investigations targeting cardiovascular pathologies. Nevertheless, future experiments are needed to optimize flow conditions in the MHS and further verify its suitability for cell culture applications.

## Supplementary Information

Below is the link to the electronic supplementary material.

Supplementary file 1: Figure S1 Compliance chamber calibration. A: Different compliance chamber air volumes ranging from 5 mL, 10 mL and 15 mL in compliance chamber C1 to up to 50 mL in compliance chamber C2 were tested in a perfusion circuit consisting of pump P1 and Bioreactor B1 at a mean pressure of 100 mmHg. To exclude potential bias due to varying vessel compliances, a non-compliant PVC-tube was implemented into the bioreactor for this pre-test. Power regression gives an estimated correlation described by the following formula: amplitude = 372.89 × air volume^−0.831^ (*R*^2^ = 0.9865). B: Exemplary pressure waveform obtained for an compliance chamber air volume of 10 ml. Due to non-compliance of the PVC-tube, multiple system-inherent reflection waves are present.(TIF 533 kb)

Supplementary file 2: Figure S2 Pressure waveform variation. Overlay of brachial artery pressure waveforms obtained from eight consecutive cycles of the same artery (A) or from single cycles of five different brachial arteries (B). *σ* = variance of the dicrotic wave maximum *t* = time, *P* = pressure (TIF 607 kb)
