# Supplementary figures and images for: Establishment of a Modular Hemodynamic Simulator for Accurate In Vitro Simulation of Physiological and Pathological Pressure Waveforms in Native and Bioartificial Blood Vessels
Source: Cardiovasc Eng Technol. 2021 Sep 23;13(2):291–306. doi: 10.1007/s13239-021-00577-0 (PMC9114050; doi:10.1007/s13239-021-00577-0)

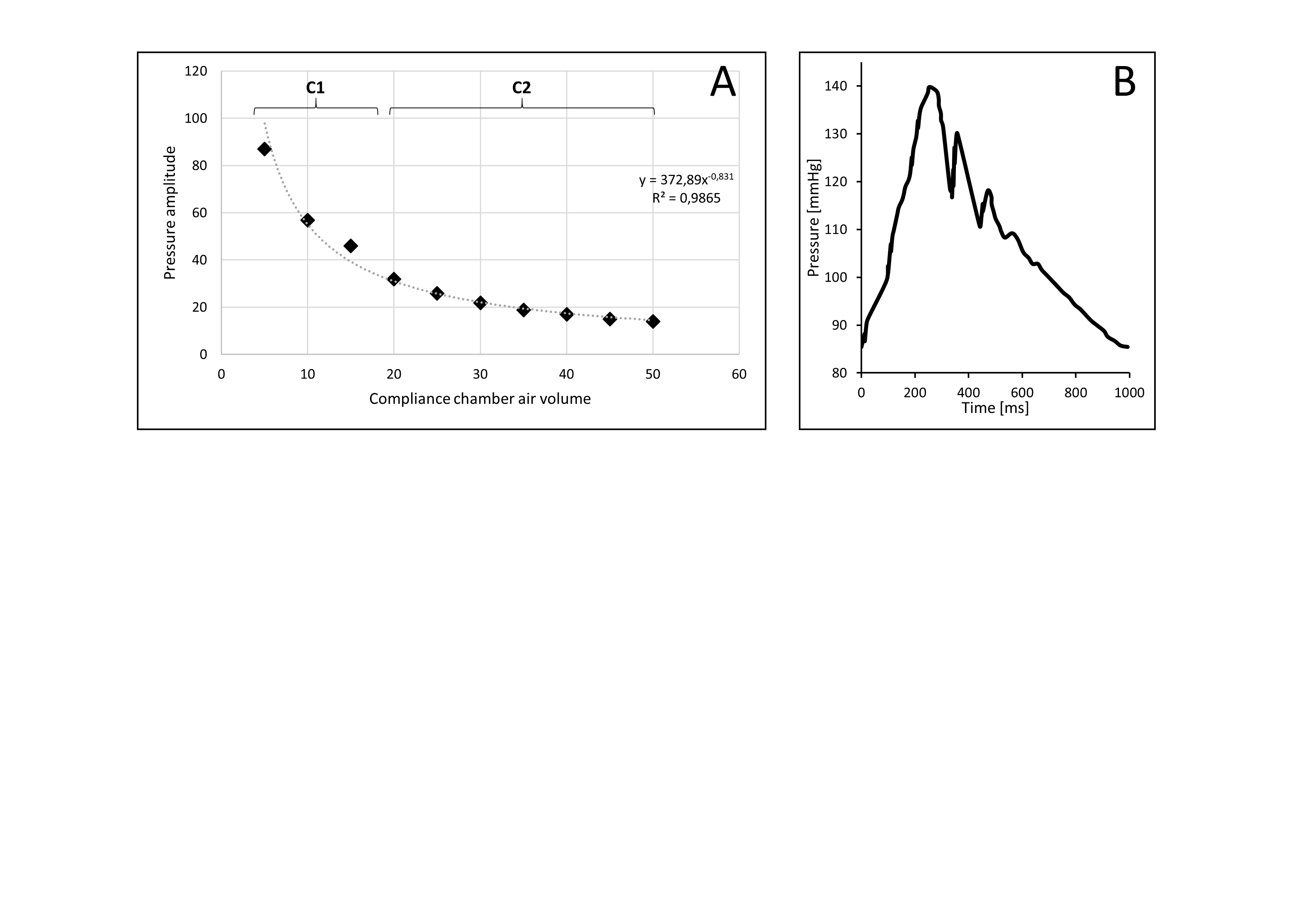

Supplement: Supplementary file 1 — Figure S1 Compliance chamber calibration. A: Different compliance chamber air volumes ranging from 5 mL, 10 mL and 15 mL in compliance chamber C1 to up to 50 mL in compliance chamber C2 were tested in a perfusion circuit consisting of pump P1 and Bioreactor B1 at a mean pressure of 100 mmHg. To exclude potential bias due to varying vessel compliances, a non-compliant PVC-tube was implemented into the bioreactor for this pre-test. Power regression gives an estimated correlation described by the following formula: amplitude = 372.89 × air volume−0.831 (R2 = 0.9865). B: Exemplary pressure waveform obtained for an compliance chamber air volume of 10 ml. Due to non-compliance of the PVC-tube, multiple system-inherent reflection waves are present.(TIF 533 kb) [file 13239_2021_577_MOESM1_ESM.tif]

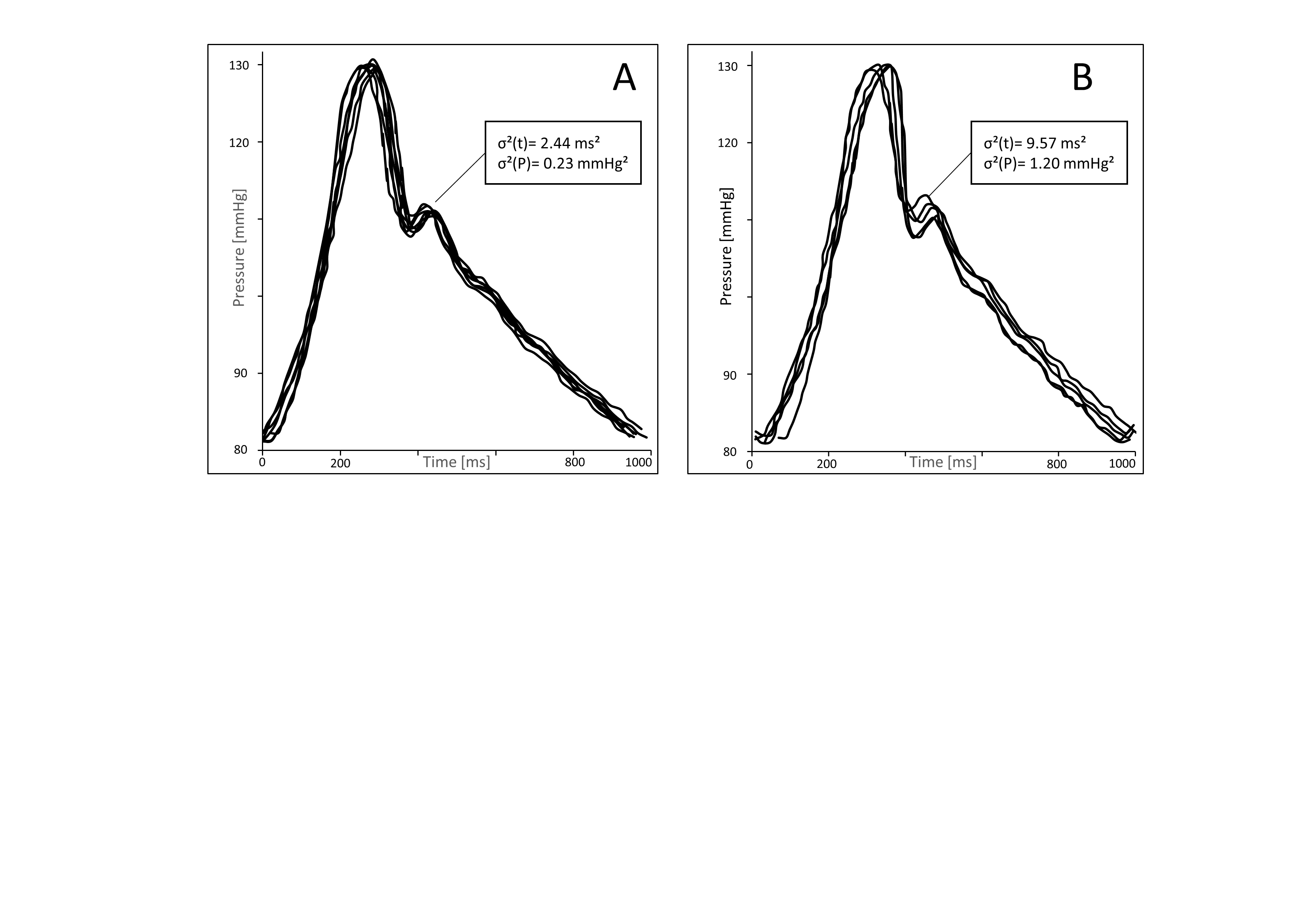

Supplement: Supplementary file 2 — Figure S2 Pressure waveform variation. Overlay of brachial artery pressure waveforms obtained from eight consecutive cycles of the same artery (A) or from single cycles of five different brachial arteries (B). σ = variance of the dicrotic wave maximum t = time, P = pressure (TIF 607 kb) [file 13239_2021_577_MOESM2_ESM.tif]
